# Supplementary material for: Hypoxic-Inflammatory Responses under Acute Hypoxia: In Vitro Experiments and Prospective Observational Expedition Trial
Source: Int J Mol Sci. 2020 Feb 4;21(3):1034. doi: 10.3390/ijms21031034 (PMC7037641; doi:10.3390/ijms21031034)
Supplement: Supplementary file 1 [file ijms-21-01034-s001.pdf]

Article

Hypoxic-Inflammatory Responses under Acute Hypoxia: In Vitro Experiments and Prospective Observational Expedition Trial

Kammerer T et al.

1. Supplemental Material

Procedure for PBMC stimulation using supernatant from previously stimulated cells (condition “CD3/CD28” of the in vitro part)

CD3/CD28 stimulation leads to non-specific, inflammatory T cell activation, which corresponds to the activation by antigen-presenting cells. Dynabeads™ Human T-Activator CD3/CD28 (Thermo Fisher Scientific, Vilnius, Lithuania) were used to simulate this T cell activation in vitro. These are magnetically charged, have a size like antigen-presenting cells and have monoclonal antibodies on their surface against the CD3 and CD28 molecules on human T cells. First, 700 µl RPMI medium was mixed with 300 µl Dynabeads™. Using the magnetic field of the QuadroMACSTM separator (Miltenyi Biotec GmbH, Bergisch Gladbach, Germany), the entire medium could be removed again without the Dynabeads™, which corresponds to a first washing step. Finally, 300 µl RPMI medium was added to the Dynabeads™. Then 10 ml RPMI medium and 10 x 106 PBMCs were pipetted into several cell culture bottles (CORNING® Flask 25 cm2, Sigma-Aldrich Chemie GmbH, Munich, Germany). 50 µl of the Dynabeads™ mixture described above was then added to each of the bottles. This was followed by a 24-hour incubation period (at 21% O_2, 5% CO_2, 37 ° C). This was followed by centrifugation (5 min, 2800 rpm), whereby the stimulated cells settled as a pellet and the "stimulation medium" was pipetted off and preserved at -80 ° C. For CD3 / CD28 stimulation in cell culture, 3x106 of the test person PBMCs were used together with 1 ml of RPMI medium and 1 ml of the "stimulation medium" as described above.
